# Survivin (BIRC5) Peptide Vaccine in the 4T1 Murine Mammary Tumor Model: A Potential Neoadjuvant T Cell Immunotherapy for Triple Negative Breast Cancer: A Preliminary Study

**DOI:** 10.3390/vaccines11030644

**Published:** 2023-03-13

**Authors:** Scott R. Burkholz, Charles V. Herst, Richard T. Carback, Paul E. Harris, Reid M. Rubsamen

**Affiliations:** 1 Flow Pharma Inc., Warrensville Heights, OH 44128, USA; sburkholz@flowpharma.com (S.R.B.); cvherst@flowpharma.com (C.V.H.); rcarback@flowpharma.com (R.T.C.);; 2 Vagelos College of Physicians and Surgeons, Columbia University, New York, NY 10032, USA; 3 Cleveland Medical Center, University Hospitals, Cleveland, OH 44106, USA; 4 Case Western Reserve School of Medicine, Cleveland, OH 44106, USA

**Keywords:** breast cancer, immunotherapy, bioinformatics, next-generation sequencing, neoadjuvant therapy, tumor-associated antigen, survivin, BIRC5

## Abstract

A triple negative breast cancer model using the murine 4T1 tumor cell line was used to explore the efficacy of an adjuvanted survivin peptide microparticle vaccine using tumor growth as the outcome metric. We first performed tumor cell dose titration studies to determine a tumor cell dose that resulted in sufficient tumor takes but allowed multiple serial measurements of tumor volumes, yet with minimal morbidity/mortality within the study period. Later, in a second cohort of mice, the survivin peptide microparticle vaccine was administered via intraperitoneal injection at the study start with a second dose given 14 days later. An orthotopic injection of 4T1 cells into the mammary tissue was performed on the same day as the administration of the second vaccine dose. The mice were followed for up to 41 days with subcutaneous measurements of tumor volume made every 3–4 days. Vaccination with survivin peptides was associated with a peptide antigen-specific gamma interferon enzyme-linked immunosorbent spot response in the murine splenocyte population but was absent from the control microparticle group. At the end of the study, we found that vaccination with adjuvanted survivin peptide microparticles resulted in statistically significant slower primary tumor growth rates in BALB/c mice challenged with 4T1 cells relative to the control peptideless vaccination group. These studies suggest that T cell immunotherapy specifically targeting survivin might be an applicable neoadjuvant immunotherapy therapy for triple negative breast cancer. More preclinical studies and clinical trials are needed to explore this concept further.

## 1. Introduction

The survivin (SVN), also known as BIRC5, protein is an inhibitor of apoptosis and is overexpressed in many malignancies, including breast cancer stem cells and breast tumor tissue [1], relative to adjacent normal adult cells and tissues (reviewed in [2,3]). These observations suggest that SVN might be an ideal tumor cell target. In fact, the National Cancer Institute declared SVN as a research priority more than twenty years ago [4]. Since then, SVN as a target of humoral and cellular immunity has been studied in animal models and in cancer patients. The structure of many immunogenic B cell and T cell epitopes of SVN have been characterized [2,5,6,7,8] and applied to the development of synthetic survivin peptide cancer vaccines. These studies have provided proof of principle data supporting several currently active human immunotherapy/vaccine clinical trials targeting SVN in glioblastoma, neuroendocrine tumors, ovarian cancer, and hormone-receptor-positive breast cancers (e.g., NCT05163080, NCT02334865, NCT04895761). In glioblastoma, immunotherapy targeting survivin has provided significant prolongation of overall survival compared with standard radiation therapy and chemotherapy [9].

Estrogen-, progesterone-receptor-negative human epidermal growth factor 2 (HER2) “negative” breast cancer (also known as triple negative breast cancer (TNBC)) accounts for up to 15–20% of all breast malignancies and has a generally worse prognosis and fewer treatment options relative to more common forms of breast cancer. Current standard of care treatment options for recurrent TNBC have been limited to conventional cytotoxic chemotherapeutic agents due to the lack of expression of molecular targets found on the more common types of breast malignancies. Adjuvant immunotherapy for TNBC is currently being explored and the approaches fall into several diverse categories (reviewed in [10]). One approach, recently approved by the Food and Drug Administration (FDA), combines chemotherapy with immune checkpoint inhibitors (ICI) with the goal of boosting the existing adaptive immune response to breast tumor antigens. However, immunohistochemistry studies show that programmed cell death ligand 1 protein (PD-L1) expression, one target of immune checkpoint inhibition therapy, is limited to about 20% of TNBC [11]. Clinical and histochemical data have confirmed the efficacy of anti-PD-L1 monoclonal antibodies (mAbs) ICI as a neoadjuvant and adjuvant treatment for TNBC patients whose tumors and/or stromal cells (including tumor-infiltrating lymphocytes) express PD-L1. While the PD-L1 positive subgroup show better overall survival, the PD-L1 negative subgroup faired equally poorly regardless of anti-PD-L1 mAb treatment [12], suggesting that additional approaches to adjuvant immunotherapy need exploration.

Adjuvant vaccine-based immunotherapy of TBNC has been recently reviewed [13]. Out of 42 clinical studies reviewed, only one limited exploratory human study [14] has targeted SVN in TBNC. With the goal of developing novel immunotherapies for TNBC, we turned to the 4T1 murine model of TNBC [15,16,17,18,19] to evaluate the therapeutic potential of a synthetic SVN-peptide-based microparticle vaccine for the adjuvant immunotherapy of TNBC. We previously developed an adjuvanted synthetic peptide poly-copolymer (lactic-co-glycolic acid) (PLGA) microparticle vaccine platform [20] capable of inducing robust therapeutic CD8+ cytotoxic T lymphocyte responses to viral antigens presented by major histocompatibility complex (MHC) Class I molecules in non-human primate [21] and murine models [22]. In this preclinical study, we report on the effects of vaccination with adjuvanted survivin peptide-loaded microspheres on orthotopically implanted tumor growth in a 4T1 murine model of triple negative breast cancer.

## 2. Materials and Methods

### 2.1. Characterization of Survivin Expression in 4T1 Cells and Normal Mouse Mammary Tissue

To better characterize survivin expression in the triple negative breast cancer model murine 4T1 cell line (American Type Culture Collection, Manassas, VA, USA), frozen 4T1 cell samples were sent to Complete Genomics (Beijing, China) for sequencing. DNA was extracted from the snap-frozen cryopreserved 4T1 cell line and BALB/c mouse tails. Sample DNA was prepared using the Agilent SureSelect XT Mouse All Exon Kit (Agilent Technologies, Santa Clara, CA, USA). RNA was extracted from the frozen 4T1 cell line as well as from snap-frozen normal mammary tissue samples harvested from BALB/c mice. RNA samples were prepared for mRNA sequencing via poly-A tail capture with MGI Tech Company reagents (MGI, Shenzhen, China). The samples were sequenced by Complete Genomics on the BGISEQ-500 (BGI, Beijing, China) at 100 base-pair paired end-reads. DNA read coverage for normal tissue and tumor was 100× and 300×, respectively, with the RNA read count at 80 million paired end-reads. To process the data, FASTP was used to perform quality control and adapter trimming, and Burrows-Wheeler maximal exact matches (BWA-MEM) aligned the reads to the GRCm38 reference mouse genome [23,24,25,26]. The germline sequences were confirmed to match the peptides identified for vaccine administration. RNA expression of survivin was examined by adapter trimming and quality control with FASTP [23], followed by pseudoalignment with Kallisto on the GENCODE v25 mouse transcriptome for quantification in Sleuth expressed as transcripts per million [25].

### 2.2. Vaccine Design/Peptide Selection

The overall strategy and rationale for the selection of synthetic peptides used to stimulate potential CTL immune responses to SVN targets presented by murine 4T1 cells has been previously described [20,22,27]. Briefly, we identified survivin peptide antigens potentially capable of stimulating MHC Class I and Class II restricted tumor-specific T cell response in BALB/c mice. These peptide sequences were determined by reviewing previous publications describing SVN-specific MHC Class I restricted T cell responses using various peptide vaccine formulations and web-based in silico predictive computational methods [28,29,30]. In addition, NetMHCIIpan and NetMHCII [30] were utilized to identify QP19, a region predicted to bind to Class II MHC to stimulate CD4 helper T cells. The sequences of these murine MHC Class I H-2K, D, and L binding peptides and murine Class II H-2 I-E and I-A binding peptides are given in Table 1.

### 2.3. Vaccine Manufacture

A blend of poly (lactic-co-glycolic acid) microspheres, prepared as described in a previous publication [21], was manufactured containing individual synthetic good manufacturing practice (GMP)-grade peptides (Peptides International, Louisville, KY, USA) selected from the primary amino acid sequence of the murine survivin protein (Table 1). The adjuvanted microsphere vaccine formulation contained 3 μg/mg of survivin-specific murine H-2K^d^ and H-2D^d^ Class 1 and H2-I-A^d^ and H2-I-E^d^ Class II peptides, and 0.5 μg/mg of the toll-like receptor 9 (TLR-9) oligonucleotide agonist, CpG (ODN-1018) (Trilink Biosciences, San Diego, CA, USA). The microspheres, approximately 6 microns in diameter, were delivered in a 200 μL volume of phosphate-buffered saline/polyoxyethylene (20) sorbitan monolaurate (Tween 20) (0.01% *v*/*v*) injectate solution containing the toll-like receptor 4 (TLR-4) agonist, monophosphoryl-lipid A (MPLA) (Avanti Polar Lipids, Alabaster, AL, USA) at a concentration of 100 μg/mL.

### 2.4. Post Vaccination Evaluation of Peripheral T Cell Responses

In the BALB/c model, 4T1 cells alone are poorly immunogenic [35]. To assess whether intraperitoneal vaccination with SVN-peptide-loaded adjuvanted microspheres evoked a SVN-peptide-specific peripheral T cell response, enzyme-linked immunosorbent spot for interferon gamma (γIFN) enzyme-linked immunosorbent spot (ELISpot) assays were performed with BALB/c splenocytes obtained from control and vaccinated surviving tumor-bearing mice on day 41 after 4T1 tumor inoculation. Splenocytes were prepared as previously described [36]. The peptide antigens used in the γIFN ELISpot assays were the same as those used in the peptide vaccine and individually added to γIFN ELISpot wells at 10 μg/mL final concentration. Triplicate wells for each respondent splenocyte population were plated. γIFN ELISpot assay plates were prepared and processed as per the manufacturer’s instructions (3321-4HPT-10, Mabtech Inc., Cincinnati, OH, USA). ELISpots were enumerated by machine (CTL S6 Entry M2, Shaker Heights, Cleveland, OH, USA) to calculate the frequency of gamma-interferon-producing T cells in the splenocyte populations. The average number of spots per well was calculated and the average well-spot count was normalized to 10^6^ splenocytes.

### 2.5. Animal Immunizations

**Figure 1:**
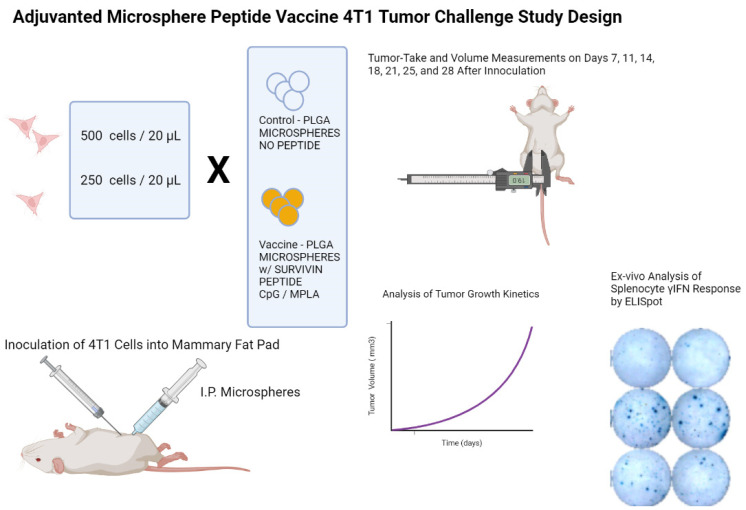
Legend. Study design schematic for a BALB/c mouse model 4T1 TNBC tumor challenge microsphere peptide vaccine efficacy study. Briefly, female BALB/c mice received an intraperitoneal injection of microsphere vaccine with or without survivin-MHC Class I-matched peptides. Two weeks later, mice received orthotopic inoculations of 250 or 500 4T1 mammary tumor cells and the second intraperitoneal dose of microparticles. Tumor volume measurements were performed every three to four days until the end of study. Following euthanasia of mice, spleens were harvested and splenocytes prepared to assess peripheral T cell reactivity to the same peptides used to prepare the microspheres. T cell immunoreactivity was visualized by elaboration of murine gamma interferon using an ELISpot assay and machine-assisted counting of the spots. The frequency of T cells producing γIFN following recognition of the MHC Class I presented immunizing peptides was then calculated.

Previous studies with the adjuvanted microsphere platform have shown that T cell expansion capable of providing protection against viral challenge is present 14 days in mouse models [20,22]. A 4T1 inoculation dose ranging study was undertaken in female BALB/c mice (20–25 gms body weight at 6–8 weeks of age) to find the maximum 4T1 cell count that would show limited tumor growth during the study and allow for an evaluation of the vaccine’s possible efficacy, as shown in the study design schematic in Supplementary Materials Figure S1. Ten mice per cell group dose were used. Based on the 4T1 dose ranging data shown in Supplementary Materials Figure S2, the challenge study was designed with one cohort receiving a 250 4T1 cell inoculation dose and the second cohort receiving 500 cells of orthotopically injected 4T1 breast cancer cells. Ten of the animals in each cohort received two doses of intraperitoneally delivered adjuvanted peptide microspheres (2.5 mg/dose), and the control mice were given two doses of blank microspheres (2.5 mg/dose, control microspheres; without peptide antigen and adjuvants) both fourteen days before 4T1 cell inoculation and again at the same time as the orthotopic injection of 4T1 cells. Subcutaneous tumor volumes were measured every three to four days with a 41 day endpoint after implantation as shown in the study design schematic in Figure 1.

Mouse tumor volume was measured non-invasively every three to four days with a micrometer applied to the tumor growing in the subcutaneous space. The tumor volume (TV) was expressed in mm^3^ using the modified ellipsoid formula: TV = ½ (length × width^2^) [37,38]. This data were used to calculate the tumor growth rate expressed as Δ mm^3^/day. Tumor-take frequencies were defined as the number of mice with measurable tumors on the indicated day divided by the total number of mice inoculated with 4T1 cells.

## 3. Results

**Figure 2:**
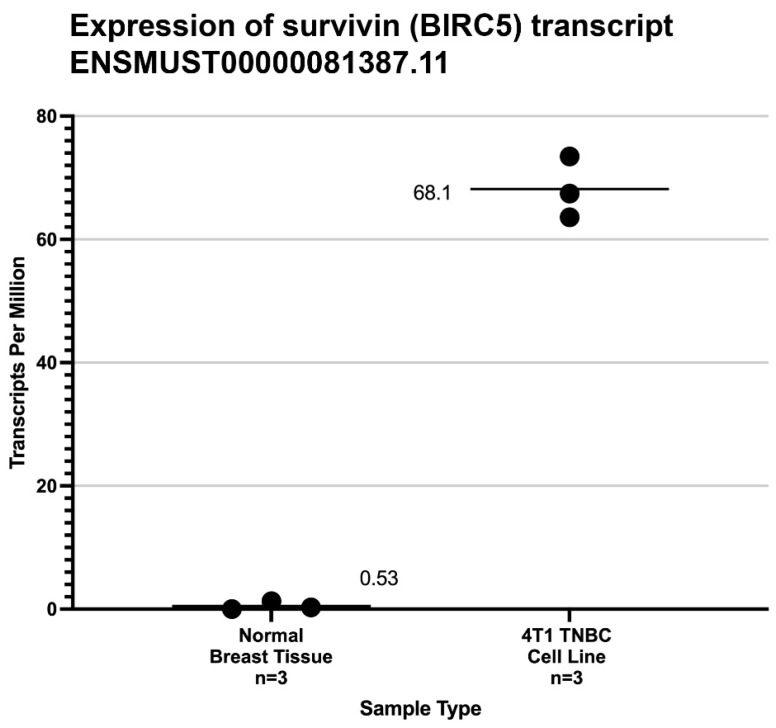
Legend. Expression level from mRNA-Seq studies of mouse wild-type survivin transcripts (ENST0000081387) [39] in normal breast tissue and the 4T1 cell line used in this study. The mean transcripts per million (TPM) are shown by black bars. The significance of the difference between the average TPM found in normal murine breast tissue and 4T1 cells was calculated by the method of Student.

Wild-type survivin messenger RNA was highly expressed in all 4T1 cell line samples studied and was seen only at very low background transcription levels in the BALB/c normal mammary tissue samples (Figure 2).

**Figure 3:**
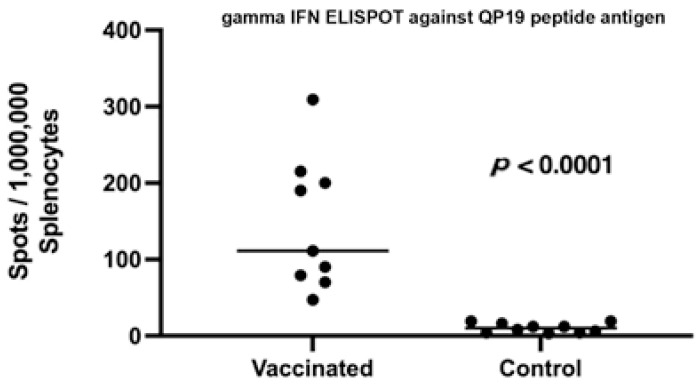
Legend. ELISpot response to QP19 in splenocytes harvested from vaccinated and unvaccinated mice that received 250 cells of 4T1. Statistical significance of the difference in the average number of ELISpots in vaccinated and control groups was determined using the unpaired, non-parametric, Mann–Whitney *t*-test.

Of the six peptides loaded into the adjuvanted microsphere formulation shown in Table 1, only QP19 (QIWQLYLKNYRIATFKNWP), produced a positive ELISpot response as shown in Figure 3. The mice who were not vaccinated did not produce a detectable response to the survivin QP19 peptide antigen. Although published studies suggested that the administered MHC Class I peptide epitopes are immunogenic in BALB/c mice, we observed that only QP19 produced a T cell response as measured by ELISpot.

**Figure 4:**
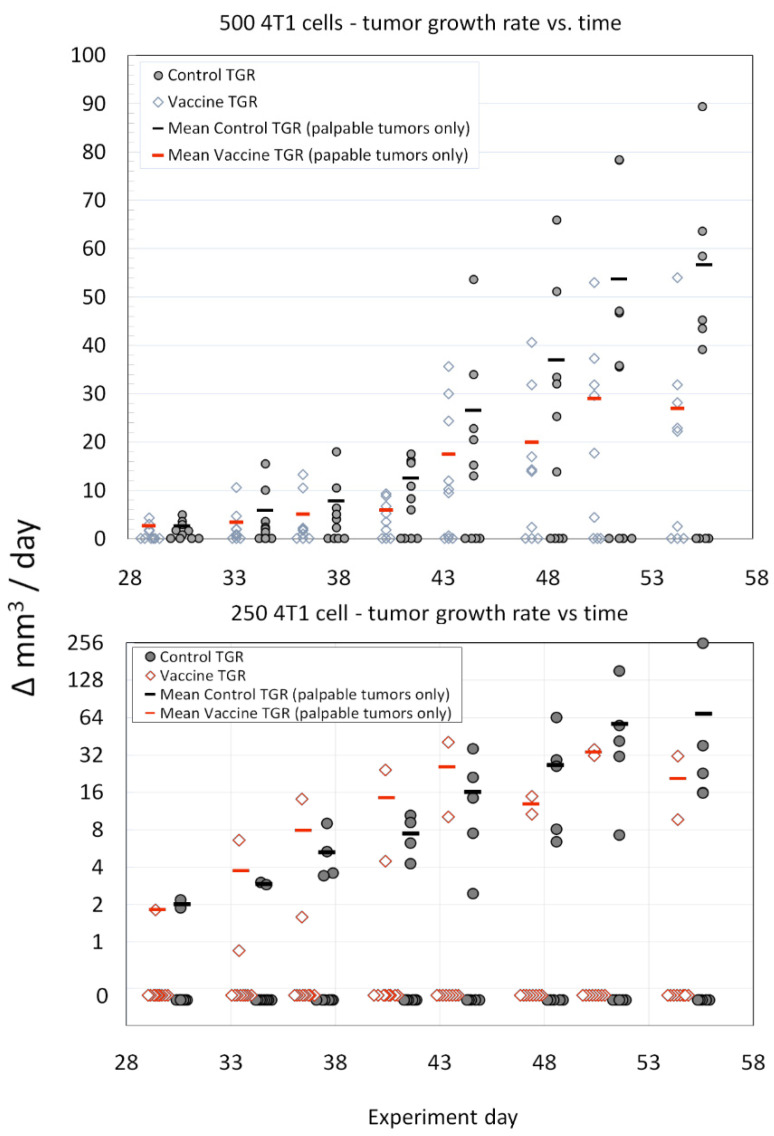
Legend. Comparison of average tumor growth rates (TGR) (vaccinated versus control) for the 500 cell and 250 cell 4T1 tumor inoculation challenge groups. The sample size was *n* = 10 for each group (open diamonds—SVN-microsphere-vaccinated mice, filled circles—control mice). The mean tumor growth rates (red bars—SVN-microsphere-vaccinated mice, black bars—control mice) were calculated from growing tumors only. The statistical significance of the differences in growth rates (only non-zero data was considered) for each tumor inoculum size and treatment group (i.e., control vs. SVN peptide microsphere) was assessed using the ANCOVA with Tukey’s honestly significant difference test (HSD) and Excel software according to the general linear model with time treated as a covariate. For either experiment, 500 cells or 250 cells, treatment (vaccination or control) was the significant variable (*p* < 0.0001 and *p* < 0.009, respectively) at the 95% confidence interval.

The inoculation dose of 4T1 at both the 250 and 500 cell levels did not result in a tumor-take frequency of 100%, but were similar to previous reports [40] and the tumor-take frequencies measured in the dose ranging study (Figure S2). However, vaccination with survivin peptide antigens was associated with statistically significant slower primary 4T1 mammary tumor growth rates compared to tumors in control mice (Figure 4). This effect was particularly evident in the 500 4T1 cells dose group, but only at later time points/tumor volumes in the 250 4T1 cell inoculum dose group. We observed γ IFN ELISpot responses to peptide QP19 in 9/10 vaccinated mice and noted that of these 10 mice, only two mice developed growing tumors.

## 4. Discussion

The application of HLA-Class I binding survivin peptides to evoke a T-cell-mediated immune response to kill tumor cells is of particular interest. Survivin (BIRC5) is normally expressed during fetal development, yet expression all but disappears in adult tissue. In adulthood, the dysregulated expression of survivin promotes tumor cell growth due to its effects on multiple signaling pathways and the inhibition of apoptosis [41]. MHC-restricted responses to peptides located within the primary amino acid sequence of the survivin protein have been shown to elicit an immune response, including in immunotherapies targeting survivin in a number of clinical trials [4]. These observations suggest that human immune central tolerance to survivin is incomplete or absent, possibly due to survivin expression before the development of operant central tolerance machinery at the thymic epithelium and medulla. It is documented that immune reactivity to certain tumor antigens represent selective pressures that can drive antigen loss and tumor escape from surveillance [42]. In the case of survivin, selective pressures leading to survivin antigen loss would theoretically lead to lower “fitness” of the remaining tumor cell population and perhaps result in a therapeutic benefit.

A comprehensive list of various peptide vaccination approaches used clinically to elicit a patient immune response against survivin-expressing tumors is shown in Table S1 (Supplementary Materials). A collection of HLA-restricted survivin peptide antigens identified across these various studies also raises the possibility of a broadly applicable immunotherapy for tumors that express survivin. As these studies also illustrate, eliciting a reliable, clinically significant immune response to peptide antigens is challenging.

Ensuring that the correct peptide sequence is selected and delivered effectively for T cell expansion to occur have been obstacles to the development of safe and effective targeted immunotherapies. For example, small peptides injected on their own, even when combined with adjuvants known to enhance a T cell response, have not been shown to trigger a particularly robust T cell response [20]. As we describe here, microspheres can be manufactured that encapsulate potentially immunogenic SVN peptides and the TLR-9 agonist, CpG, in a biodegradable PLGA polymer, delivered after reconstitution in a saline solution with the TLR-4 agonist, MPLA, by intraperitoneal injection to produce a cellular immune response to the administered peptide antigens, as demonstrated by ELISpot [20].

In this study, we found that multiple putative peptide antigens, derived from the primary sequence of survivin and predicted to bind to the MHC Class I molecules of BALB/c mice, did not elicit a detectable ex-vivo immune response. The QP19 peptide antigen was predicted to bind to I-A^d^/I-E^d^, the MHC Class II molecules of BALB/c; however, it elicited an ex-vivo gamma-IFN T cell ELISpot response and this response was associated with slower tumor growth rates. This observation suggests that protective T cell responses were vaccine-induced and operant during tumor growth.

One possible explanation for this observation would be the presence of one or more CD8+ T cell epitopes co-localized within the QP19 nineteen-mer, producing the observed T cell response and anti-tumor growth activity associated with vaccination. Analysis of all possible overlapping peptides of 8–9 amino acids in length within QP19 using NetMHC and NetMHCpan found four potential BALB/c-MHC-matched peptide antigens as listed in Table S2 [30,31]. An alternative, but not mutually exclusive, hypothesis is that the QP19 peptide presented by MHC Class II on antigen-presenting cells provided significant CD4+ T cell help to promote the expansion of CD8+ T cells recognizing naturally processed and presented survivin MHC Class I epitopes. The identification of possible survivin-protective CD8+ T cell epitopes demonstrated by ex-vivo ELISpot response and the formal demonstration of CD4+ T cell helper activity evoked by vaccination with QP19 await further experimentation.

The survivin protein wild-type isoform (~140 amino acids long) represents a source of over 130 possible murine MHC Class I molecules binding 9 mer peptides, more than twice that amount if: (a) both the BALB/c H-2K^d^ and H-2D^d^ MHC molecules and (b) MHC binding peptides may be as short as 8 mers or as long as 10 mers, are considered. Not all of these possible peptides will bind to the MHC Class I molecules for reasons of structural incompatibility or they are not abundant enough because of barriers in antigen processing. Nevertheless, there may be at least dozens of potentially immunogenic peptides in the remaining pool. In vivo, however, only a few CD8+ T cell responses are reproducibly detectable in the initial phase of an immune response. These peptide epitopes are called immunodominant [43]. In later stages of the T cell response to a particular protein antigen, as the response to immunodominant epitopes wanes, a second wave of CD8+ T cell responses to subdominant peptide epitopes may take place. The recruitment and expansion of the T cell responses to subdominant epitopes may be critical in the delay of tumor progression [44,45].

If such CD8+ T cell epitopes within QP19 do indeed exist, they may be immunodominant relative to the other MHC Class I peptide SVN antigens used in these experiments. Immunodominant CD8+ T clones may prevail over subdominant clones, masking their response. The use of monoclonal antibodies recognizing programed cell death protein 1 (anti-PD-1 mAbs) has been shown to promote epitope spreading to subdominant epitopes in the context of anti-tumor CD8+ T cell responses [41,46], and certainly suggests a potential role for immune checkpoint inhibitors in SVN peptide microsphere vaccination for triple negative breast cancer immunotherapy.

We note a number of other limitations to the current study. While we did document slower tumor growth and ELISpot reactivity to the immunizing survivin peptides tested in this study, future experiments in this model should include a demonstration of the presence of tumor-infiltrating lymphocytes presumably responsible for the effects on tumor growth. These experiments might include multiplex immunohistochemistry studies (e.g., staining with anti-CD3, anti-CD8, and anti-CD4 monoclonal antibodies) of the tumor tissue itself. Alternatively, the mRNA expression profile of the bulk tumor tissue of vaccinated and control mice could be captured by next-generation-sequencing and compared, with particular attention paid to the quantitation of known markers of a T-cell-inflamed phenotype (i.e., IL-6, chemokines, T cell markers, and a type I IFN signature).

This preliminary study of immunotherapy of TNBC using an adjuvanted PLGA microparticle platform only included five survivin peptides. The MHC-peptide-binding prediction algorithms used in these studies suggest there may be a number of other SVN-derived peptides that bind to the H2-K^d^ and H2-D^d^ molecules expressed by BALB/c-antigen-presenting cells. Similarly, the number of different synthetic peptides tested in human clinical trials (Supplementary Materials Table S1) is limited. In the context of adjuvant immunotherapy of TNBC, it seems worthwhile to expand the repertoire of human immunogenic survivin peptide antigens, both dominant and subdominant, to increase the probability of provoking a protective immune response in patients following microparticle vaccination.

The use of immunotherapy for breast cancer has gained attention recently [42,47]. Tumor-associated antigens, in contrast to neoantigens, provide the opportunity to develop immunotherapy targeting a fixed set of peptide epitopes with collective human leucocyte antigen (HLA) restrictions predicted to provide broad population coverage that could be administered to breast cancer patients without the need for patient-specific tumor gene sequencing and manufacturing of personalized immunotherapy.

## 5. Conclusions

Targeted T cell immunotherapy triggering a cytotoxic T cell immune response against survivin as neoadjuvant therapy has the potential to reduce tumor recurrence and the metastatic spread after surgical excision of the primary breast tumor if the number of cells remaining after tumor debulking is low enough to allow a CTL attack that is sufficiently vigorous to blunt tumor-take and tumor growth rates. Previous studies have seen mixed efficacy with unprotected peptides used as immunotherapy [4,43,48].

Short peptides, such as those we have selected for this study, are typically inefficient at evoking MHC Class I-restricted CD8+ T cell responses. The poor immunogenicity of peptide antigens [44,49], administered as neat peptides, is likely due to a number of interdependent factors, including in vivo degradation, poor peptide uptake by inactivated antigen-presenting cells, poor expression of MHC Class I and II molecules, costimulatory molecules on antigen-presenting cells, and poor cross-presentation by dendritic cell type antigen-presenting cells [45,50]. The microencapsulation of SVN peptides in adjuvanted PLGA microspheres appears to circumvent many of the above impediments to provoke an anti-tumor T cell response in the 4T1 mouse breast cancer model (Supplementary Figure S3). A delivery system, such as the adjuvanted microsphere encapsulation described herein, may be able to effectively deliver peptides to produce T cell expansion against tumor-associated antigen targets such as survivin expressed by TNBC patients.

## Figures and Tables

**Table 1:** Legend. Literature references and computational prediction tools supporting a BALB/c MHC match to each of the six peptides microencapsulated into the adjuvanted microsphere vaccine platform described here. An acronym for each peptide, created from the first and last amino acid residue in the peptide sequence, and the number of residues between them was created and is given in the column, “Epitope Name”. The proposed restriction element to which the peptide binds is given in the “MHC Class” column and the position of the peptide with the wild-type survivin protein isoform is supplied in the adjacent columns. Lastly, the reference to the predictive algorithm used in peptide selection is supplied.

Epitope Name	Peptide Sequence	MHC Class	Position	MHC ^1^ Match Screening Method
AL9	ATFKNWPFL	I	20–28	[30,31]
AM9	AFLTVKKQM	I	85–93	[29]
GI9	GWEPDDNPI	I	66–74	[28,29]
TI9	TAKTTRQSI	I	127–135	[29]
QP19	QIWQLYLKNYRIATFKNWP	I/II	8–26	[30,32]
PADRE	AKFVAAWTLKAAA	II	N/A	[33,34]

^1^ MHC, Major Histocomaptibilty Complex.

## Data Availability

DNA and RNA seq data can be accessed at NIH SRA under accession # PRJNA868747.

## Supplementary Materials

The following supporting information can be downloaded at: https://www.mdpi.com/article/10.3390/vaccines11030644/s1. Table S1: Clinical trial list detailing survivin peptide immunotherapy studies conducted over the last two decades for a variety of tumor types [51,52,53,54,55,56,57,58,59]. Table S2: NetMHC and NetMHCpan were used to scan all possible overlapping peptides of 8–9 amino acids in length within QP19 [30,31,32]. Figure S1: Study design schematic for 4T1 TNBC cell line inoculation dose escalation study. Figure S2: Average growth rate curves shown after injection of escalating numbers of 4T1 TNBC cancer cells into BALB/c mouse mammary tissue at T0. Figure S3: Proposed mechanism of action of survivin-peptide loaded, adjuvanted poly lactide-co-glycolide (PLGA) microspheres enhance immunogenicity of H2K and H2D tailored nonameric synthetic peptides to evoke a 4T1 tumor specific cytotoxic T cell response.
