# Supplementary material for: Survivin (BIRC5) Peptide Vaccine in the 4T1 Murine Mammary Tumor Model: A Potential Neoadjuvant T Cell Immunotherapy for Triple Negative Breast Cancer: A Preliminary Study
Source: Vaccines (Basel). 2023 Mar 13;11(3):644. doi: 10.3390/vaccines11030644 (PMC10051918; doi:10.3390/vaccines11030644)
Supplement: Supplementary file 1 [file vaccines-11-00644-s001.zip › vaccines-2230483-supplementary.pdf]

Supplementary Materials

Table S1. Legend. Clinical trial list detailing survivin peptide immunotherapy studies conducted over the last two decades for a variety of tumor types.

Cancer Type	Clinical Stage	Peptide	HLA	Delivery	Results	Citations
Glioblastoma	Phase II	DLAQCFFMFKELEGW	A*02, A*03, A*24	A peptide mutated from the wild type sequence conjugated to the adjuvant keyhole limpet haemocyanin, injected subcutaneous 4 times biweekly along with Montanide ISA 51 with sargramostim.	Increased survivin-specific IgG antibodies and CD8+ T-cells generated in majority of patients with some efficacy for PFS and OS along with temozolomide.	[9,51]
Multiple	Phase II	AYACNTSTL	A*24:02	Subcutaneous injection of peptide at multiple intervals, 14 days apart; addition of IFN-α or IFN-β in later studies; addition of IFA in later studies	Increased levels of peptide specific CTL in a portion of patients, but adjuvants are added due to lack of clinical efficacy, however, progression free survival	[7,52-54]

					was not increased.	
Melanoma	Phase II	LMLGEFLKL, and a peptide from IDO	A*02	Multiple subcutaneous injection of peptide mutated from survivin and indoleamine 2,3-dioxygenase with Montanide ISA 51. Topical 5% imiquimod cream and GM-CSF to assist in delivery and response.	The majority of patients had an immune response, with an increase of memory CD4+ and CD8+. No significant results seen, but tumor regression in one individual.	[55]
Solid Tumors	Phase I	FTELTLGEF, LMLGEFLKL, RISTFKNWPK, STFKNWPFL, LPPAWQPFL	A*01, A*02, A*03, A*24, B*7	Subcutaneous injection of peptide, some mutated from wild-type, multiple times with Montanide ISA 51.	Survivin-specific T-cells in the majority of patients, with most of those not detected before vaccination. Clinical results not evaluated.	[56]
Melanoma	Phase II	FTELTLGEF, LMLGEFLKL, EPDLAQCFY	A*01, A*02, B*35	Deep Subcutaneous injection of 3 peptides differing from wild type	Increase survivin-specific CD8+ T-cells in the majority of	[57]

				sequence along with Montanide ISA 51. Different regimen used for vaccination frequency and addition of cyclophosphamide.	individuals. Significant extension seen in overall survival.	
Prostate	Phase I/II	TLGEFLKLDRERAKN, TLPPAWQPFL, ELTLGEFLKL, and multiple non-survivin class 1 and class 2 peptides	DRB1*0X, A*02	Peptides subcutaneously injected or loaded onto autologous DC at intervals with one of the following: Imiquimod, GM-CSF, local hyperthermia or the TLR-7/8 agonist mRNA/protamine complex.	Several HLA-DRB1 present this peptide for a Th1 CD4+ response, however, one patient had an anaphylactic reaction to this peptide after multiple doses.	[58,59]

Table S2. Legend. NetMHC and NetMHCpan were used to scan all possible overlapping peptides of 8-9 amino acids in length within QP19 [30–32]. The four potential BALB/c MHC matched class I peptide antigens found within QP19 are listed here.

Epitope Name	Peptide Sequence	Class	Position
LI8	LYLKNYRI	1	12-19
LA9	LYLKNYRIA	1	12-20
KF8	KNYRIATF	1	15-22
LF9	LKNYRIATF	1	14-21

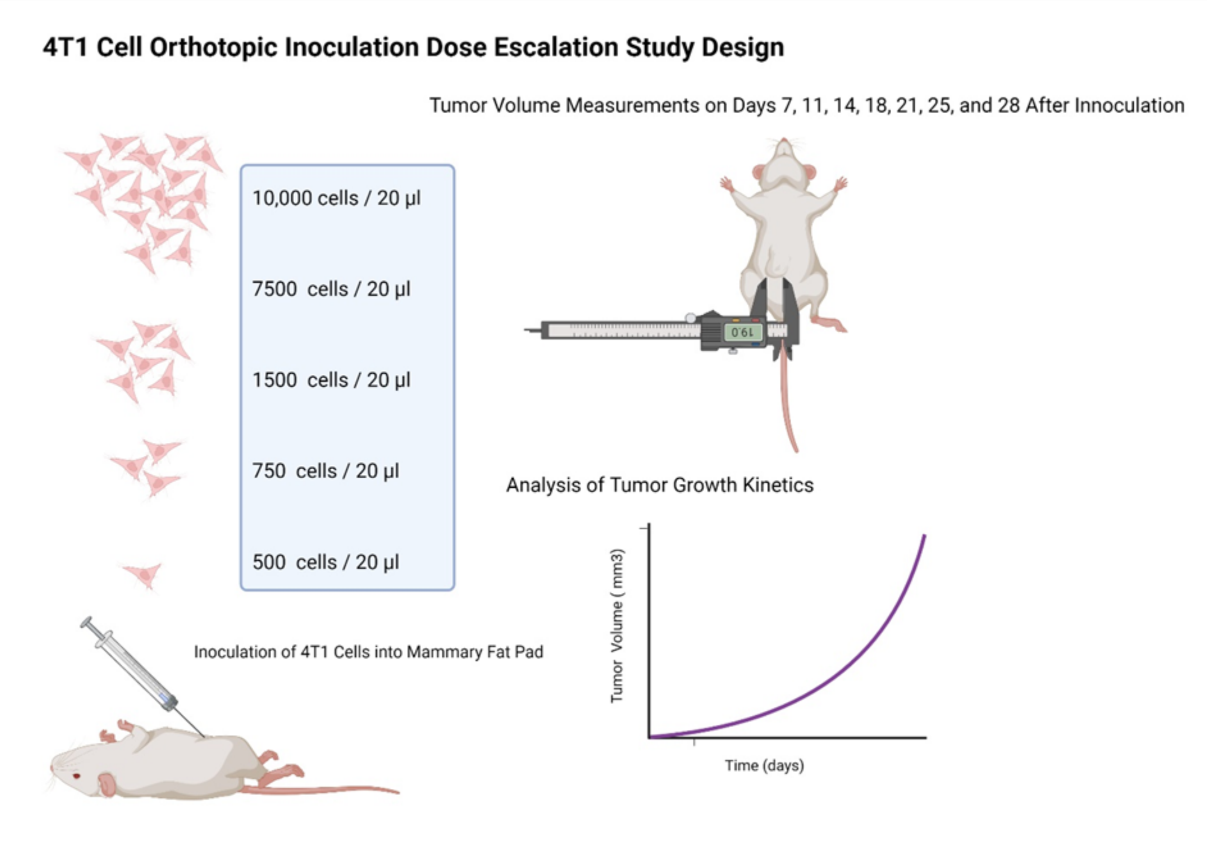

Figure S1. Legend. Study design schematic for 4T1 TNBC cell line inoculation dose escalation study. Varying numbers of 4T1 tumor cells were injected into mouse mammary tissue.

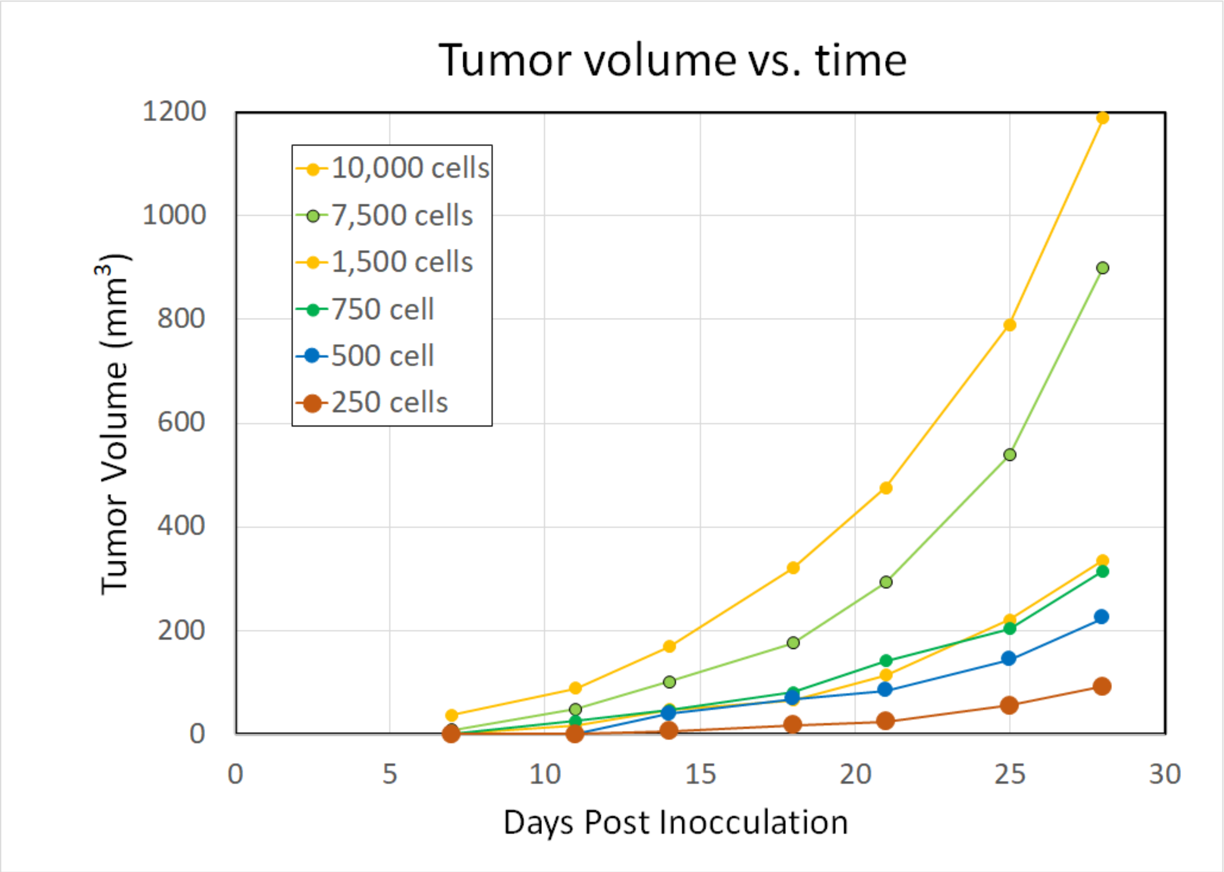

Figure S2. Legend. Average growth rate curves shown after injection of escalating numbers of 4T1 TNBC cancer cells into BALB/c mouse mammary tissue at T0. Sample size was n=10 mice per group. Overall survival during the study period was 100% for the 250, 500, and1500 4T1 cell doses. At 1500 cells/dose the survival rate dropped below 80%.

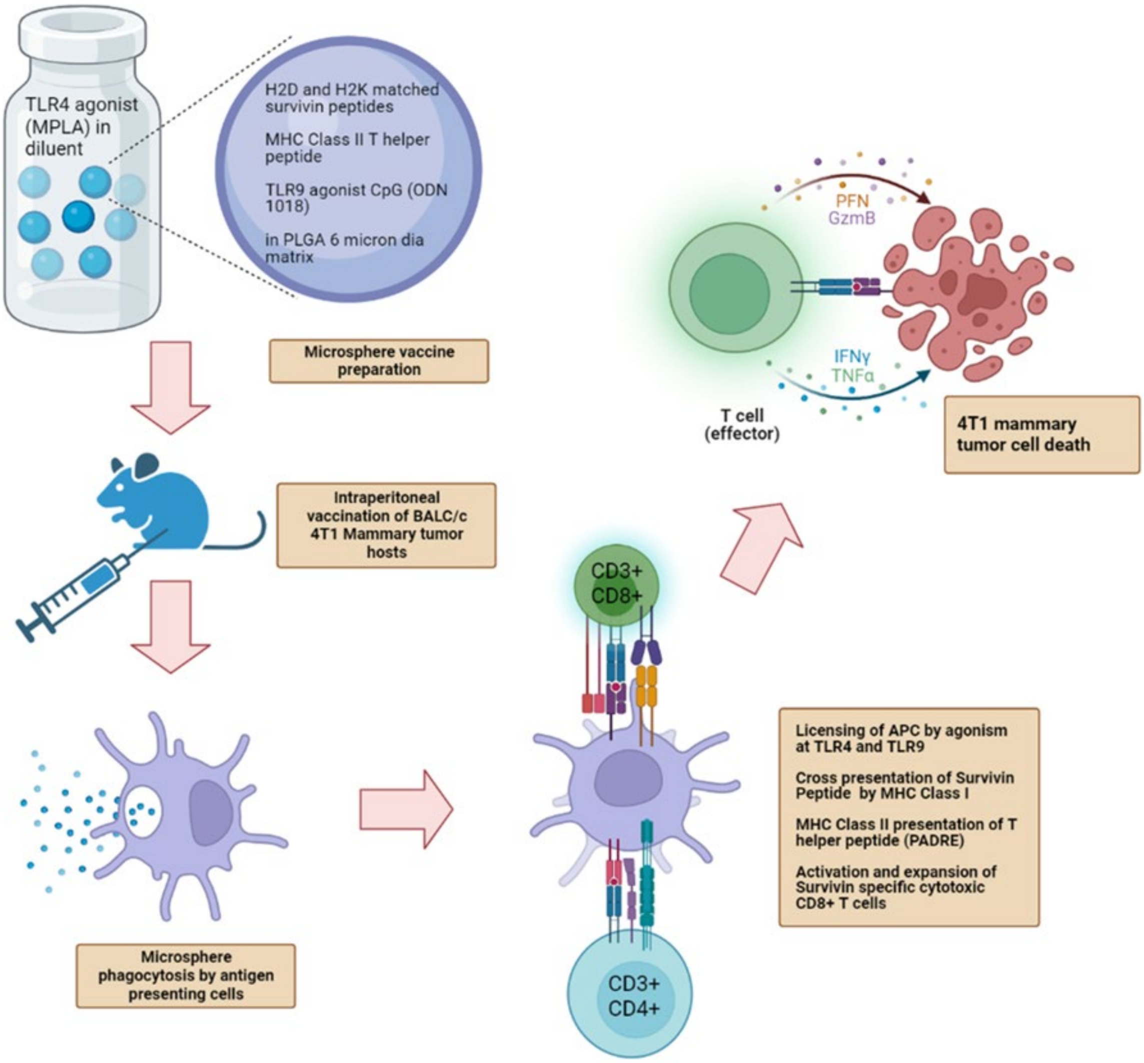

Figure S3. Legend**.** Proposed mechanism of action of survivin-peptide loaded, adjuvanted poly lactide-co-glycolide (PLGA) microspheres enhance immunogenicity of H2K and H2D tailored nonameric synthetic peptides to evoke a 4T1 tumor specific cytotoxic T cell response. Further investigation on the peptide evoked T cells response will be needed to confirm this working hypothesis. .
